# Viewpoint matters: objective performance metrics for surgeon endoscope control during robot-assisted surgery

**DOI:** 10.1007/s00464-016-5090-8

**Published:** 2016-07-15

**Authors:** Anthony. M. Jarc, Myriam J. Curet

**Affiliations:** 1 Medical Research, Intuitive Surgical, Inc., 5655 Spalding Drive, Norcross, GA 30092 USA; 2 VA Palo Alto, Stanford, CA USA

**Keywords:** Robot-assisted surgery, Surgeon training, Proficiency, Performance metrics, Endoscope control, Visualization

## Abstract

**Background:**

Effective visualization of the operative field is vital to surgical safety and education. However, additional metrics for visualization are needed to complement other common measures of surgeon proficiency, such as time or errors. Unlike other surgical modalities, robot-assisted minimally invasive surgery (RAMIS) enables data-driven feedback to trainees through measurement of camera adjustments. The purpose of this study was to validate and quantify the importance of novel camera metrics during RAMIS.

**Methods:**

New (*n* = 18), intermediate (*n* = 8), and experienced (*n* = 13) surgeons completed 25 virtual reality simulation exercises on the *da Vinci* Surgical System. Three camera metrics were computed for all exercises and compared to conventional efficiency measures.

**Results:**

Both camera metrics and efficiency metrics showed construct validity (*p* < 0.05) across most exercises (camera movement frequency 23/25, camera movement duration 22/25, camera movement interval 19/25, overall score 24/25, completion time 25/25). Camera metrics differentiated new and experienced surgeons across all tasks as well as efficiency metrics. Finally, camera metrics significantly (*p* < 0.05) correlated with completion time (camera movement frequency 21/25, camera movement duration 21/25, camera movement interval 20/25) and overall score (camera movement frequency 20/25, camera movement duration 19/25, camera movement interval 20/25) for most exercises.

**Conclusions:**

We demonstrate construct validity of novel camera metrics and correlation between camera metrics and efficiency metrics across many simulation exercises. We believe camera metrics could be used to improve RAMIS proficiency-based curricula.

Effective visualization of the operative field is essential to successful surgery. It enables surgeons to identify diseased and healthy anatomy as well as instrument–tissue interactions as they treat disease states. Poor visualization can be costly and resulted in decreased patient safety through an increase in surgical errors [1, 2]. It is critical surgeons learn to optimally visualize the operative field just as they learn to use instruments.

Different forms of surgery use different methods to visualize patient anatomy. During open surgery, surgeons trade off invasiveness for access and visualization (i.e., a larger incision allows a direct view and interaction with anatomy but is more invasive to the patient). During minimally invasive surgery (MIS), an endoscope is used to peer inside a patient through a small incision, thereby reducing invasiveness (compared to open surgery) while maintaining or even improving how well a surgeon sees anatomy. However, this imposes new skills surgeons must learn. Manual laparoscopy requires coordination between a surgeon and an assistant, where the surgeon verbally instructs the assistant where to position the endoscope since the surgeon’s hands are dedicated to instruments. Robot-assisted minimally invasive surgery (RAMIS) removes the assistant from the workflow and returns control of the endoscope to the surgeon: the surgeon uses hand controllers to switch between controlling her instruments and her camera. It is apparent from these examples that many new MIS technologies require surgeons learn how to control an endoscope in order to achieve optimal visualization.

Commonly, MIS surgeon trainees learn how to visualize the operative field by observing experienced surgeons control their endoscopes and replicating their behaviors while receiving feedback from their mentors (i.e., an apprenticeship model [3]). Alternatively, objective rating scales can be used to evaluate how well trainees visualize their environment (see robotic control, depth perception in GEARS [4]), but these face challenges in being administered given they are time-consuming and a largely manual process involving video review. Furthermore, the apprenticeship model and objective rating scales can be inefficient given they require oversight by an experienced mentor in order for a trainee to receive feedback on his performance (although crowd-sourced objective rating scales have recently shown promise [5]). More automated, objective measures of visualization performance stand to improve training efficiency by delivering feedback to trainees even without expert supervision [6].

A primary obstacle to more automated, objective measures of performance is the ability to unobtrusively measure behavior during training or even live surgery. RAMIS is the exception; surgeon behavior can be measured unobtrusively by leveraging its tele-operative architecture, offering the potential to develop automated, objective performance measures that can be used by a surgeon throughout her training [7]. Many academic teams have used these measures to validate training exercises and set proficiency guidelines [8–10], as well as to develop advanced algorithms to classify skill [11–13]. However, most performance measures focus on hand movements, instrument movements, environment interactions, or discrete errors and overlook measures specific to visualization through proficient endoscope control [14–16]. In laparoscopy, several training paradigms have been designed specifically to teach surgeons how to visualize their environment [17–19]; however, only a few performance measures focused on camera behavior have been proposed, including camera stability [20], endoscope path length [21], and horizon alignment [22]. Despite similar camera-specific exercises existing on RAMIS virtual reality simulators, objective performance measures focused specifically on endoscope control during RAMIS are lacking in virtual reality training and clinical scenarios.

In this work, we define performance metrics for endoscope control for a wide variety of existing RAMIS simulation exercises targeting many different technical skills, including endoscope control, needle driving, and instrument manipulation. We evaluate the construct validity of the newly defined metrics by comparing them between populations of novice, intermediate, and experienced RAMIS surgeons. Furthermore, we examine how well endoscope control metrics differentiate new and experienced RAMIS surgeons compared to conventional movement metrics. Finally, we offer motivation to examine these metrics clinically by correlating them to completion time, a commonly used metric to estimate proficiency in clinical procedures. In the end, we believe endoscope control metrics can improve surgeon training and ultimately visualization strategies by being incorporated into existing training protocols and proficiency standards for RAMIS trainees.

## Materials and methods

### Dataset

Study participants were enrolled in an Institutional Review Board-approved study. Thirty-nine RAMIS surgeons completed 25 simulation exercises using the *da Vinci* ^*®*^ *Skills Simulator* (dVSS) for the *da Vinci* *Si* ^*®*^ Surgical System (Intuitive Surgical Inc., Sunnyvale, CA). The exercises included: Camera Targeting—Level 1, Camera Targeting—Level 2, Dots and Needles—Level 1, Dots and Needles—Level 2, Energy Dissection—Level 1, Energy Dissection—Level 2, Energy Switching—Level 1, Match Board—Level 1, Match Board—Level 2, Match Board—Level 3, Needle Targeting, Peg Board—Level 1, Pick and Place, Ring and Rail—Level 1, Ring and Rail—Level 2, Ring Walk—Level 1, Ring Walk—Level 2, Ring Walk—Level 3, Scaling, Suture Sponge—Level 1, Suture Sponge—Level 2, Suture Sponge—Level 3, Thread the Rings, and Tubes. Participants were from multiple specialties: 11 general surgery, 16 gynecology, and 12 urology. Twenty-seven were practicing surgeons, 3 were fellows, and 9 were residents greater than PGY II. Surgeons were grouped based on expertise with 18 new, 8 intermediate, and 13 experienced RAMIS surgeons. New surgeons were defined as having completed less than 20 RAMIS procedures, intermediate surgeons between 21 and 150 RAMIS procedures, and experienced surgeons greater than 150 RAMIS procedures. New surgeons included residents, fellows, and practicing open and laparoscopic surgeons. All surgeons may have had prior experience in laparoscopic or open surgery. Each surgeon completed one trial of each exercise. The exercises were completed consecutively in a common order by all surgeons.

For each simulation exercise, kinematic and event data from the surgical system and virtual environment were recorded. The kinematic data included the movements of the hand controllers, instruments, and endoscope. The event data included all states of the *da Vinci* Surgical System, such as master clutch events, camera movement events, and head-in events, as well as select states of the virtual environment. In addition, the performance metrics and overall scores computed by the dVSS were recorded.

### Skill assessment metrics

We defined three novel performance metrics related to how surgeons control their endoscope, and as a result how they visualize their environment, during RAMIS. We call these metrics camera metrics. The first performance metric was camera movement frequency (CFrq). It was defined as the average number of endoscope movements made by a surgeon per second over the entire exercise. The second performance metric was camera movement duration (CDur). CDur was defined as the average time in seconds of all endoscope movements over the entire exercise. Finally, the third performance metric was camera movement interval (CInt). It was defined as the average time in seconds between endoscope movements over an entire exercise.

In addition, we extracted four conventional performance metrics commonly used during simulation—overall score (OverallScore), completion time (CompTime), economy of motion (EOM), and master workspace range (MWR). OverallScore was the *MScore™* used to give a single score for a given exercise by combining multiple metrics (Mimic Technologies, Inc., Seattle, WA). CompTime was defined as the total time in seconds to complete an exercise. EOM was the total distance travelled by the instruments in meters throughout an exercise. Finally, MWR was defined as 85 % of the larger of two radii in meters that represented the distance between the average hand position (in three dimensions) and each sampled position. All of these performance metrics are used in the *MScore* on the dVSS. Note that given the heterogeneity of the simulation exercises and associated errors, the comparison in this paper focused on a select few efficiency metrics while excluding other metrics related to efficiency and errors.

### Construct validity of camera metrics

We defined construct validity as the ability of the performance metrics to differentiate populations of surgeons with varying expertise. In particular, we compared the mean performance of new, intermediate, and experienced surgeons for each camera metric as well as the overall score and completion time. Student’s *t* tests were used to determine significance (*p* < 0.05).

### Camera and conventional metric comparisons

The ability of camera metrics to differentiate new and experienced surgeons across all exercises was compared to the subset of conventional metrics (see “Skill assessment metrics” section). First, the mean of performance metrics for each exercise was normalized across exercises according to Eq. (1):

1 $$x_{i}^{n} = \frac{{\left( {x_{i} - x_{\hbox{min} } } \right)}}{{\left( {x_{\hbox{max} } - x_{\hbox{min} } } \right)}}$$

*x*_min_ and *x*_max_ are the minimum and maximum, respectively, of the mean performance metrics for each exercise, *x*_*i*_ is the mean performance metric for exercise *i*, and *x*_*i*_^*n*^ is the normalized mean performance metric for exercise *i*. Next, the differences between the normalized mean performances of novice and experienced surgeons across all exercises were computed according to Eq. (2):

2 $$d = \left| {\left( {\mu_{1} - \mu_{2} } \right)} \right|$$

*d* is the mean difference, *μ*_1_ and *μ*_2_ are the mean of the normalized metrics across all exercises for two groups (i.e., new and experienced surgeons), and |·| represents the absolute value. The mean differences of normalized metrics were sorted in decreasing magnitude to illustrate their ability to differentiate new and experienced performance. A Student’s *t* test was used to make pair-wise comparisons across camera and conventional performance metrics (*p* < 0.05).

### Correlation to conventional performance metrics

The correlation of camera metrics with metrics typically used to assess clinical performance was used to examine whether camera metrics could be good candidates to include in assessments of clinical performance. The correlation coefficient assuming a linear model was computed between each camera metric and both CompTime and OverallScore while including new, intermediate, and experienced surgeon data. A Student’s *t* test was used to determine significance (*p* < 0.05).

## Results

**Fig. 1:**
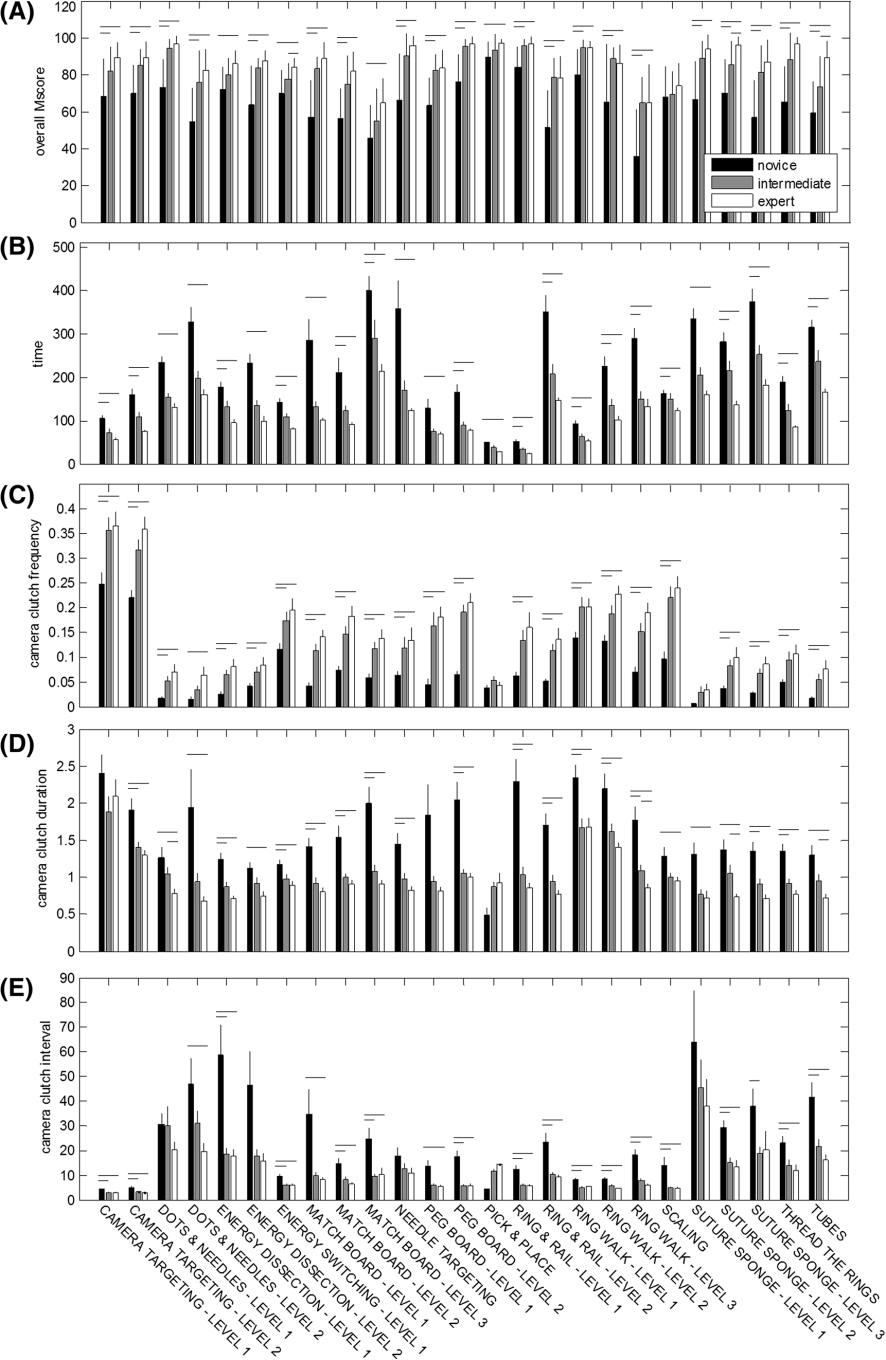
Construct validity of conventional metrics and camera metrics. **A** Overall score, **B** completion time, **C** camera movement frequency (CFrq), **D** camera movement duration (CDur), and **E** camera movement interval (CInt). *Horizontal bars* indicate significant differences between surgeon groups (*p* < 0.05)

Bar plots for OverallScore, CompTime, and all three camera metrics across all simulation exercises are shown in Fig. 1. Tables 1, 2, and 3 list the results from the *t* tests comparing the camera metric means for new, intermediate, and experienced RAMIS surgeons. Across all exercises except Scaling, experienced surgeons achieved a significantly higher OverallScore than new surgeons. Similarly, intermediate surgeons achieved a significantly higher OverallScore than new surgeons for 20/25 exercises. In three exercises—Energy Switching—Level 1, Suture Sponge—Level 2, and Tubes—experienced surgeons achieved a significantly higher OverallScore than intermediate surgeons.

**Table 1:** Mean comparisons and correlation coefficients for camera movement frequency (CFrq) across all simulation exercises

	New	Intermed.	Exp.	*p* value (N vs. I)	*p* value (N vs. E)	*p* value (I vs. E)	Time		Overall score	
							Corr. coef.	*p* value	Corr. coef.	*p* value
Camera Targeting—Level 1	0.25 ± 0.10	0.36 ± 0.10	0.37 ± 0.11	**0.0051**	**0.0044**	0.8392	0.6081	**<0.0001**	0.4958	**0.0007**
Camera Targeting—Level 2	0.22 ± 0.07	0.32 ± 0.07	0.36 ± 0.09	**0.0009**	**<0.0001**	0.2132	0.7381	**<0.0001**	0.6359	**<0.0001**
Dots and Needles—Level 1	0.02 ± 0.02	0.05 ± 0.04	0.07 ± 0.05	**0.0095**	**0.0048**	0.3155	0.6457	**<0.0001**	0.5722	**0.0003**
Dots and Needles—Level 2	0.01 ± 0.02	0.03 ± 0.03	0.06 ± 0.06	0.1296	**0.0429**	0.1151	0.4483	**0.0114**	0.2947	0.1075
Energy Dissection—Level 1	0.03 ± 0.02	0.06 ± 0.04	0.08 ± 0.05	**0.0008**	**0.0006**	0.3565	0.5366	**0.0003**	0.4945	**0.0010**
Energy Dissection—Level 2	0.04 ± 0.03	0.07 ± 0.04	0.08 ± 0.05	**0.0295**	**0.0114**	0.4572	0.2531	0.1151	0.2114	0.1904
Energy Switching—Level 1	0.12 ± 0.05	0.17 ± 0.07	0.20 ± 0.08	**0.0113**	**0.0039**	0.4945	0.4388	**0.0036**	0.4499	**0.0028**
Match Board—Level 1	0.04 ± 0.03	0.11 ± 0.05	0.14 ± 0.05	**<0.0001**	**<0.0001**	0.1888	0.7673	**<0.0001**	0.6571	**<0.0001**
Match Board—Level 2	0.07 ± 0.04	0.15 ± 0.07	0.18 ± 0.07	**0.0046**	**0.0002**	0.2226	0.6424	**<0.0001**	0.5270	**0.0011**
Matchboard—Level 3	0.06 ± 0.04	0.12 ± 0.05	0.14 ± 0.07	**0.0005**	**0.0003**	0.4152	0.7567	**<0.0001**	0.6611	**<0.0001**
Needle Targeting	0.06 ± 0.04	0.12 ± 0.08	0.13 ± 0.09	**0.0233**	**0.0061**	0.6404	0.3254	**0.0355**	0.3044	**0.0500**
Peg Board—Level 1	0.04 ± 0.05	0.16 ± 0.10	0.18 ± 0.08	**0.0020**	**0.0001**	0.6706	0.3493	0.0501	0.3624	**0.0415**
Peg Board—Level 2	0.06 ± 0.03	0.19 ± 0.06	0.21 ± 0.07	**<0.0001**	**<0.0001**	0.4591	0.6932	**<0.0001**	0.6356	**<0.0001**
Pick and Place	0.04 ± 0.03	0.05 ± 0.03	0.04 ± 0.02	0.3775	0.6933	0.4804	0.1181	0.6200	−0.0394	0.8692
Ring and Rail—Level 1	0.06 ± 0.03	0.13 ± 0.08	0.16 ± 0.12	**0.0014**	**0.0019**	0.5150	0.6409	**<0.0001**	0.4581	**0.0018**
Ring and Rail—Level 2	0.05 ± 0.03	0.11 ± 0.05	0.14 ± 0.08	**0.0002**	**0.0002**	0.3582	0.6256	**<0.0001**	0.5331	**0.0002**
Ring Walk—Level 1	0.14 ± 0.06	0.20 ± 0.08	0.20 ± 0.06	**0.0155**	**0.0065**	0.9668	0.6207	**<0.0001**	0.4959	**0.0007**
Ring Walk—Level 2	0.13 ± 0.05	0.19 ± 0.06	0.23 ± 0.06	**0.0099**	**<0.0001**	0.1230	0.7449	**<0.0001**	0.4580	**0.0020**
Ring Walk—Level 3	0.07 ± 0.04	0.15 ± 0.06	0.19 ± 0.08	**0.0002**	**<0.0001**	0.1769	0.7885	**<0.0001**	0.5099	**0.0006**
Scaling	0.10 ± 0.07	0.22 ± 0.08	0.24 ± 0.09	**<0.0001**	**<0.0001**	0.5702	0.1325	0.4028	−0.3042	0.0501
Suture Sponge—Level 1	0.01 ± 0.00	0.03 ± 0.04	0.03 ± 0.04	0.2101	0.1332	0.8011	0.4389	**0.0362**	0.3530	0.0985
Suture Sponge—Level 2	0.04 ± 0.02	0.08 ± 0.05	0.10 ± 0.07	**0.0015**	**0.0015**	0.4202	0.5657	**0.0001**	0.4509	**0.0031**
Suture Sponge—Level 3	0.03 ± 0.02	0.07 ± 0.04	0.09 ± 0.05	**0.0009**	**0.0002**	0.3024	0.4876	**0.0011**	0.5585	**0.0001**
Thread the Rings	0.05 ± 0.03	0.09 ± 0.06	0.11 ± 0.06	**0.0114**	**0.0020**	0.6108	0.4264	**0.0049**	0.4244	**0.0051**
Tubes	0.02 ± 0.01	0.05 ± 0.05	0.08 ± 0.07	**0.0035**	**0.0018**	0.3661	0.5165	**0.0005**	0.4828	**0.0014**

Significant comparisons (*p* < 0.05) are highlighted in bold

**Table 2:** Mean comparisons and correlation coefficients for camera movement duration (CDur) across all simulation exercises

	New	Intermed.	Exp.	*p* value (N vs. I)	*p* value (N vs. E)	*p* value (I vs. E)	Time		Overall score	
							Corr. coef.	*p* value	Corr. coef.	*p* value
Camera Targeting—Level 1	2.40 ± 1.09	1.89 ± 0.75	2.09 ± 0.84	0.1601	0.4062	0.5212	−0.2594	0.0931	−0.1886	0.2258
Camera Targeting—Level 2	1.91 ± 0.65	1.40 ± 0.30	1.30 ± 0.26	**0.0143**	**0.0035**	0.3699	−0.5880	**<0.0001**	−0.5925	**<0.0001**
Dots and Needles—Level 1	1.27 ± 0.58	1.05 ± 0.34	0.78 ± 0.23	0.2607	**0.0132**	**0.0324**	−0.5928	**0.0001**	−0.3638	**0.0292**
Dots and Needles—Level 2	1.94 ± 1.81	0.94 ± 0.41	0.68 ± 0.21	0.0676	**0.0327**	0.0633	−0.3425	0.0593	−0.1825	0.3259
Energy Dissection—Level 1	1.24 ± 0.39	0.87 ± 0.25	0.71 ± 0.18	**0.0067**	**0.0002**	0.0789	−0.4239	**0.0057**	−0.2254	0.1566
Energy Dissection—Level 2	1.12 ± 0.33	0.92 ± 0.27	0.75 ± 0.22	0.0882	**0.0024**	0.0929	−0.5038	**0.0009**	−0.4861	**0.0015**
Energy Switching—Level 1	1.17 ± 0.27	0.97 ± 0.25	0.89 ± 0.22	**0.0490**	**0.0062**	0.3880	−0.5365	**0.0003**	−0.4628	**0.0023**
Match Board—Level 1	1.42 ± 0.46	0.91 ± 0.30	0.80 ± 0.23	**0.0035**	**0.0003**	0.3118	−0.6846	**<0.0001**	−0.5432	**0.0004**
Match Board—Level 2	1.54 ± 0.67	1.00 ± 0.21	0.91 ± 0.18	**0.0175**	**0.0044**	0.2910	−0.5752	**0.0003**	−0.4859	**0.0031**
Matchboard—Level 3	2.00 ± 0.94	1.08 ± 0.32	0.90 ± 0.21	**0.0032**	**0.0003**	0.1026	−0.5209	**0.0004**	−0.3815	**0.0127**
Needle Targeting	1.45 ± 0.58	0.98 ± 0.28	0.82 ± 0.21	**0.0112**	**0.0012**	0.1234	−0.6561	**<0.0001**	−0.5669	**<0.0001**
Peg Board—Level 1	1.84 ± 1.73	0.94 ± 0.32	0.81 ± 0.20	0.1042	0.0779	0.2971	−0.4919	**0.0042**	−0.4002	**0.0232**
Peg Board—Level 2	2.05 ± 0.99	1.05 ± 0.21	1.00 ± 0.21	**0.0020**	**0.0008**	0.5314	−0.6704	**<0.0001**	−0.7447	**<0.0001**
Pick and Place	0.49 ± 0.45	0.87 ± 0.23	0.93 ± 0.44	0.0871	0.0882	0.7885	0.1381	0.5615	0.1513	0.5243
Ring and Rail—Level 1	2.29 ± 1.33	1.03 ± 0.40	0.86 ± 0.24	**0.0027**	**0.0006**	0.1745	−0.5503	**0.0001**	−0.3942	**0.0081**
Ring and Rail—Level 2	1.70 ± 0.67	0.94 ± 0.31	0.77 ± 0.20	**0.0008**	**<0.0001**	0.0964	−0.6639	**<0.0001**	−0.5869	**<0.0001**
Ring Walk—Level 1	2.35 ± 0.74	1.66 ± 0.48	1.68 ± 0.42	**0.0076**	**0.0079**	0.9028	−0.4826	**0.0010**	−0.4300	**0.0040**
Ring Walk—Level 2	2.20 ± 0.86	1.62 ± 0.38	1.40 ± 0.28	**0.0339**	**0.0047**	0.1102	−0.5457	**0.0002**	−0.2502	0.1100
Ring Walk—Level 3	1.77 ± 0.74	1.09 ± 0.32	0.86 ± 0.21	**0.0049**	**0.0002**	**0.0449**	−0.6292	**<0.0001**	−0.4202	**0.0062**
Scaling	1.29 ± 0.51	0.99 ± 0.25	0.95 ± 0.22	0.0652	**0.0416**	0.6669	−0.1298	0.4127	0.1129	0.4766
Suture Sponge—Level 1	1.31 ± 0.66	0.77 ± 0.25	0.72 ± 0.36	0.0508	**0.0421**	0.7679	−0.5922	**0.0029**	−0.3949	**0.0622**
Suture Sponge—Level 2	1.36 ± 0.62	1.05 ± 0.42	0.73 ± 0.15	0.1355	**0.0019**	**0.0187**	−0.5779	**<0.0001**	−0.4376	**0.0042**
Suture Sponge—Level 3	1.36 ± 0.53	0.91 ± 0.28	0.71 ± 0.18	**0.0094**	**0.0004**	0.0512	−0.6565	**<0.0001**	−0.6601	**<0.0001**
Thread the Rings	1.36 ± 0.38	0.92 ± 0.25	0.76 ± 0.22	**0.0014**	**<0.0001**	0.1067	−0.6612	**<0.0001**	−0.5324	**0.0003**
Tubes	1.30 ± 0.56	0.96 ± 0.31	0.72 ± 0.17	0.0620	**0.0022**	**0.0321**	−0.5437	**0.0002**	−0.3995	**0.0097**

Significant comparisons (*p* < 0.05) are highlighted in bold

**Table 3:** Mean comparisons and correlation coefficients for camera movement intervals (CInt) across all simulation exercises

	New	Intermed.	Exp.	*p* value (N vs. I)	*p* value (N vs. E)	*p* value (I vs. E)	Time		Overall score	
							Corr. coef.	*p* value	Corr. coef.	*p* value
Camera Targeting—Level 1	4.42 ± 1.64	2.98 ± 0.94	3.01 ± 1.06	**0.0089**	**0.0121**	0.9409	−0.5254	**0.0003**	−0.4769	**0.0012**
Camera Targeting—Level 2	5.01 ± 2.61	3.19 ± 0.72	2.86 ± 0.62	**0.0213**	**0.0072**	0.2261	−0.5914	**<0.0001**	−0.6318	**<0.0001**
Dots and Needles—Level 1	30.59 ± 18.86	29.80 ± 28.96	20.33 ± 11.50	0.9493	0.1559	0.3015	−0.3356	0.0604	−0.2810	0.1193
Dots and Needles—Level 2	47.04 ± 35.73	31.11 ± 17.89	19.42 ± 11.57	0.2618	**0.0497**	0.1133	−0.7061	**0.0001**	−0.5917	**0.0023**
Energy Dissection—Level 1	58.77 ± 51.49	18.49 ± 9.01	17.72 ± 10.16	**0.0102**	**0.0122**	0.8431	−0.3421	**0.0330**	−0.4413	**0.0049**
Energy Dissection—Level 2	46.46 ± 55.68	17.72 ± 10.51	15.66 ± 11.16	0.0789	0.0846	0.6470	−0.2299	0.1591	−0.2325	0.1543
Energy Switching—Level 1	9.60 ± 4.61	6.06 ± 2.10	5.94 ± 2.90	**0.0160**	**0.0223**	0.9068	−0.3597	**0.0193**	−0.4618	**0.0021**
Match Board—Level 1	34.91 ± 41.74	9.99 ± 4.83	8.22 ± 4.07	0.0519	**0.0382**	0.3417	−0.3740	**0.0246**	−0.3734	**0.0249**
Match Board—Level 2	14.80 ± 8.63	8.24 ± 4.15	6.37 ± 2.48	**0.0343**	**0.0039**	0.1984	−0.6241	**<0.0001**	−0.6240	**<0.0001**
Matchboard—Level 3	24.81 ± 19.38	9.36 ± 4.14	10.37 ± 9.24	**0.0117**	**0.0198**	0.7301	−0.5080	**0.0006**	−0.4461	**0.0031**
Needle Targeting	17.69 ± 14.57	12.64 ± 7.75	11.07 ± 7.08	0.2684	0.1582	0.6024	−0.2528	0.1063	−0.4117	**0.0067**
Peg Board—Level 1	13.72 ± 10.42	6.00 ± 2.68	5.54 ± 1.79	0.0504	**0.0354**	0.6753	−0.5376	**0.0067**	−0.6100	**0.0016**
Peg Board—Level 2	17.65 ± 10.06	5.74 ± 1.80	5.75 ± 3.53	**0.0004**	**0.0004**	0.9913	−0.6202	**<0.0001**	−0.7285	**<0.0001**
Pick and Place	4.35 ± 1.74	11.61 ± 3.14	14.40 ± 0.00	0.0633	0.1329	0.5232	0.2227	0.6714	0.2512	0.6311
Ring and Rail—Level 1	12.24 ± 7.30	6.14 ± 1.85	5.67 ± 2.07	**0.0092**	**0.0110**	0.5861	−0.5208	**0.0008**	−0.6320	**<0.0001**
Ring and Rail—Level 2	23.24 ± 16.90	10.17 ± 3.99	9.03 ± 4.32	**0.0111**	**0.0064**	0.4903	−0.5144	**0.0004**	−0.5815	**<0.0001**
Ring Walk—Level 1	8.11 ± 3.50	5.17 ± 1.53	5.22 ± 1.47	**0.0087**	**0.0093**	0.9402	−0.5911	**<0.0001**	−0.5098	**0.0005**
Ring Walk—Level 2	8.36 ± 2.91	5.82 ± 1.83	4.75 ± 1.30	**0.0101**	**0.0003**	0.0976	−0.6958	**<0.0001**	−0.4354	**0.0035**
Ring Walk—Level 3	18.15 ± 9.49	7.92 ± 2.94	6.10 ± 2.26	**0.0009**	**0.0001**	0.0900	−0.5730	**<0.0001**	−0.3806	**0.0129**
Scaling	13.88 ± 15.03	4.88 ± 1.51	4.82 ± 1.62	**0.0412**	**0.0486**	0.9287	−0.0769	0.6329	0.2046	0.1995
Suture Sponge—Level 1	63.83 ± 87.60	45.64 ± 40.59	37.95 ± 37.77	0.6641	0.5034	0.7303	−0.4894	**0.0462**	−0.6828	**0.0025**
Suture Sponge—Level 2	29.19 ± 13.24	14.98 ± 7.56	13.27 ± 9.25	**0.0021**	**0.0016**	0.6172	−0.4445	**0.0041**	−0.3987	**0.0108**
Suture Sponge—Level 3	37.81 ± 30.46	18.73 ± 10.73	20.30 ± 26.50	**0.0409**	0.1240	0.8456	−0.3917	**0.0113**	−0.4650	**0.0022**
Thread the Rings	23.05 ± 11.98	13.90 ± 9.06	11.90 ± 8.12	**0.0295**	**0.0094**	0.5679	−0.4313	**0.0044**	−0.2673	0.0871
Tubes	41.60 ± 25.24	21.47 ± 12.10	16.10 ± 8.55	**0.0169**	**0.0032**	0.2169	−0.4978	**0.0017**	−0.4637	**0.0038**

Significant comparisons (*p* < 0.05) are highlighted in bold

Experienced surgeons performed all exercises significantly faster than new surgeons. Intermediate surgeons performed 17/25 exercises significantly faster than new surgeons. There were no significant differences in CompTime across the exercises between intermediate and experienced surgeons.

Experienced surgeons had significantly higher CFrq than new surgeons for all but two exercises—Pick and Place (*p* = 0.6933) and Suture Sponge—Level 1 (*p* = 0.1332) (Table 1). In 22/25 exercises, intermediate surgeons had significantly higher CFrq than new surgeons. There were no significant differences in CFrq between intermediate and experienced surgeons.

Experienced surgeons had significantly shorter CDur than new surgeons for all exercises except Camera Targeting—Level 1 (*p* = 0.4062), Peg Board—Level 1 (*p* = 0.0779), and Pick and Place (*p* = 0.0882) (Table 2). In 15/25 exercises, intermediate surgeons had significantly shorter CDur than new surgeons. Finally, in 4/25 exercises, experienced surgeons had significantly shorter CDur than intermediate surgeons.

In 19/25 exercises, experienced surgeons had significantly shorter CInt than new surgeons whereas intermediate surgeons had significantly shorter CInt than new surgeons in 17/25 exercises (Table 3). There were no significant differences in CInt between intermediate and experienced surgeons.

**Fig. 2:**
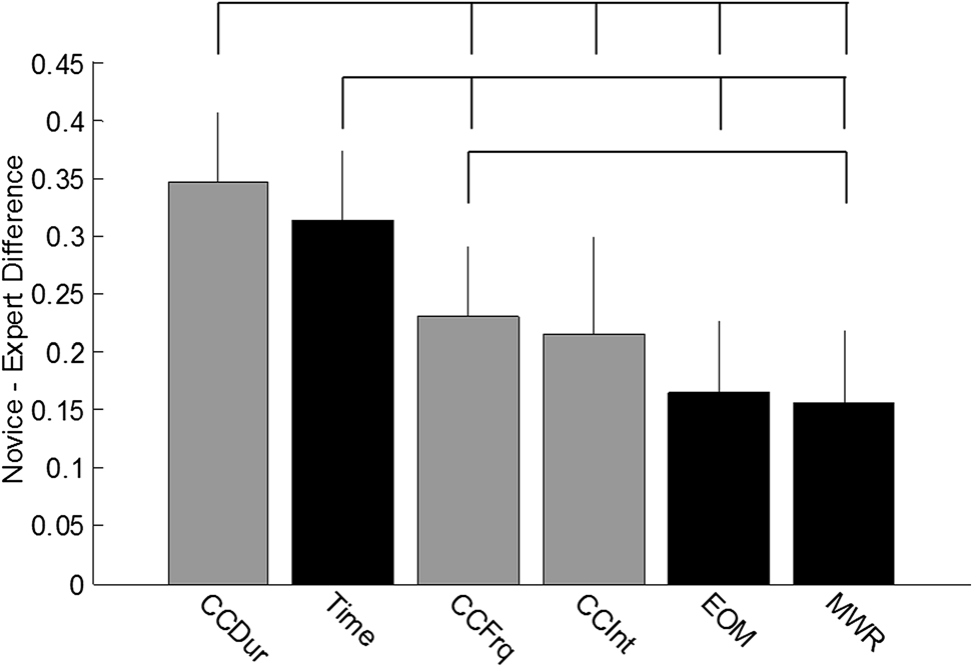
Difference between novice and expert surgeons mean normalized metrics across all exercises (ordered by magnitude). *Gray bars* correspond to viewpoint metrics. *Black bars* correspond to conventional performance metrics. *Error bars* represent +1 SD. *Top brackets* indicate significant differences between metrics (*p* < 0.05)

The mean differences of normalized metrics illustrated CDur and CompTime best differentiated new and experienced surgeons across all exercises (Fig. 2). The mean difference in CDur was significantly different than the mean difference in CFrq, CInt, EOM, and MWR. The mean difference in CompTime was significantly different than the mean difference in CFrq, EOM, and MWR. CFrq significantly differentiated experienced and new surgeons better than MWR but not EOM. The mean difference in CInt was not significantly different than the mean difference in CFrq, EOM, or MWR.

Individual metric correlations between CompTime and OverallScore are listed in Table 1 (CFrq), Table 2 (CDur), and Table 3 (CInt). CompTime was significantly correlated with CFrq in 21/25 exercises, CDur in 21/25 exercises, and CInt in 20/25 exercises. Pick and Place and Scaling did not correlate with CompTime for any camera metrics. CFrq during Energy Dissection—Level 2 (*p* = 0.1151) and Peg Board—Level 1 (*p* = 0.0501), CDur during Camera Targeting—Level 1 (*p* = 0.0931) and Dots and Needles—Level 2 (*p* = 0.0593), and CInt during Dots and Needles—Level 1 (*p* = 0.0604), Energy Dissection—Level 2 (*p* = 0.1591), and Needle Targeting—Level 1 (*p* = 0.1063) did not correlate significantly with CompTime.

OverallScore was significantly correlated with CFrq in 20/25 exercises, CDur in 19/25 exercises, and CInt in 20/25 exercises. Again, Pick and Place and Scaling did not correlate with OverallScore for any camera metrics. CFrq during Dots and Needles—Level 2 (*p* = 0.1075), Energy Dissection—Level 2 (*p* = 0.1904), and Suture Sponge—Level 1 (*p* = 0.0985), CDur during Camera Targeting—Level 1 (*p* = 0.2258), Dots and Needles—Level 2 (*p* = 0.3259), Energy Dissection—Level 1 (*p* = 0.1566), and Ring Walk—Level 2 (*p* = 0.1100), and CInt during Dots and Needles—Level 1 (*p* = 0.1193), Energy Dissection—Level 2 (*p* = 0.1543), and Thread the Rings (*p* = 0.0871) did not correlate significantly with CompTime.

## Discussion

Objective performance measures of RAMIS surgeon technical skills are critical to minimizing learning curves and maximizing patient safety [6, 23–25]. The results presented here show construct validity of new performance metrics related to endoscope control during virtual reality simulation exercises for RAMIS (Fig. 1; Tables 1, 2, 3). Similar to conventional efficiency measures (e.g., completion time and economy of motion), the camera metrics consistently differentiated new and experienced surgeons. A few consistent exceptions existed (Pick and Place and Scaling), but these exercises may not have been challenging enough for this comparison. Further metric comparisons between new or experienced surgeons and intermediate surgeons offered a window into the learning curves of each simulation exercise; those that differentiated intermediate from experienced surgeons may be more challenging than those that do not and could be used after simpler exercises and vice versa. In addition, an aggregated analysis of the camera metrics showed they differentiated new and experienced surgeons across all tasks as well as, and sometimes better than, conventional efficiency metrics (Fig. 2). Finally, camera metrics showed strong correlation between OverallScore and CompTime (a metric used to evaluate efficiency in clinical scenarios) (Tables 1, 2, 3). This suggests that camera metrics could be used to evaluate procedural performance; however, additional validation studies are needed. This result combined with the results of construct validity across most exercises suggests endoscope control is an essential underlying technical skill for many types of surgical tasks, such as camera control, *Endowrist* ^*®*^ manipulation, needle driving, and energy and dissection. Given endoscope control is intrinsically linked to effective visualization, surgeon competency defined using camera metrics could be helpful in ensuring safe and effective surgery.

Although we show that camera metrics are important indicators of RAMIS technical skill, we do not know exactly why experienced surgeons adopt the specific behavior when controlling the endoscope. Could there be optimal camera positions for specific tasks simply to assist with surveillance of the operative field? Alternatively, could camera movements be exploited by experienced surgeons to extract relevant visual information from their environment, such as depth information [26, 27] or estimates of interaction forces [28]? One hypothesis is current RAMIS systems do not include haptic feedback, and therefore, surgeons might rely on visual cues to estimate interaction forces accurately [29]. Another hypothesis is that the viewpoint influences the ease by which experienced surgeons make subsequent movements. This could be a result of better visualization as well as relative position and orientation of their instruments and the environment (e.g., anatomy and needle). Future research studies—both in controlled laboratory and applied clinical settings—should examine the underlying causes of these endoscope control behaviors so that future training scenarios and RAS technologies could be optimized to surgeon sensorimotor control.

Thorough characterization of endoscope control might also be useful for technology development. Automated and objective measures of endoscope control could be used in intelligent tutoring systems to deliver formative feedback during surgeon training [30]. Such systems have the potential to consistently remind inexperienced surgeons to optimize how they visualize patient anatomy and their instruments without requiring an expert surgeon or instructor to be present. Similarly, several research teams have developed robot arms and algorithms to give control of the endoscope to surgeons during conventional laparoscopy [31–33] and to automate control during RAMIS [34, 35]. These laparoscopic systems remove the need for the surgeon to verbally instruct an assistant how to adjust the endoscope, whereas the RAMIS systems remove the need to control the endoscope altogether. It will be imperative that these systems remain flexible enough to accommodate the sensory demands of surgeons and do not inherently limit a surgeon’s ability to optimize his view of the operative field, which could increase the likelihood of technical errors.

Several limitations exist with this research study. First, the simulation exercises are relatively simple and involved a subset of technical skills compared to an actual clinical procedure. Similarly, the simulation exercises contain different visual information than live tissue. Live tissue has soft, shiny, and whispy structures that the simulation exercises do not replicate. It would be interesting to reproduce the same camera metrics during clinical procedures where surgeons might experience familiar anatomy, surgical planning, or other cognitive demands that could influence how and why they choose a certain viewpoint of the operative field. Finally, the viewpoint measures used in this study were simply the gross positions of the endoscope. Additional examinations of surgeon viewpoint could examine specific aspects of the field of view (the extent of the observable anatomy that a surgeon sees with a particular endoscope position), point of view (the direction from which the specific anatomy within the field of view are viewed), and focal point (the specific point of interest within the view).

Despite these limitations, camera metrics might be helpful for discriminating surgeon skill or setting proficiency standards if incorporated into existing RAMIS simulation training exercises, such as the dVSS, *dV*-*Trainer* ^*®*^ (Mimic Technologies, Inc.), *RobotiX Mentor™* (3D System, Inc.), and *RoSS™* (Simulated Surgical Systems, LLC). For scenarios outside of simulation where data might not be recorded directly from a RAMIS platform, camera metrics could be further emphasized by expert trainers, proctors, and attending surgeons, possibly through supplements to existing objective rating scales. Such scenarios might include dry laboratory exercises, wet laboratory training tasks, and clinical procedures. Interestingly, it is possible to replicate the camera metrics presented here for any type of task on RAMIS platforms by extracting the icons indicating camera control that normally appear on the surgeon’s screen or by using image processing algorithms to analyze changes in viewpoint. In this way, future efforts toward automated, objective evaluation are not limited to those research teams with access to internal data from RAMIS platforms.

In the end, we show that camera metrics are compelling RAMIS surgeon performance measures, comparable to many conventional, efficiency metrics focused on time and instrument movements. We believe that they could be used to improve current RAMIS surgeon training paradigms and proficiency-based curricula. By encouraging surgical trainees to exhibit optimal endoscope control, we could continue to improve patient safety.
